# NLRP3 Activation Was Regulated by DNA Methylation Modification during *Mycobacterium tuberculosis* Infection

**DOI:** 10.1155/2016/4323281

**Published:** 2016-06-06

**Authors:** Meili Wei, Lu Wang, Tao Wu, Jun Xi, Yuze Han, Xingxiang Yang, Ding Zhang, Qiang Fang, Bikui Tang

**Affiliations:** ^1^School of Life Science, Institute of Neurobiology and Anhui Key Laboratory of Infection and Immunity, Bengbu Medical College, Bengbu 233030, China; ^2^Department of Microbiology and Parasitology, Anhui Key Laboratory of Infection and Immunity, Bengbu Medical College, Bengbu 233030, China

## Abstract

*Mycobacterium tuberculosis* (Mtb) infection activates the NLRP3 inflammasome in macrophages and dendritic cells. Much attention has been paid to the mechanisms for regulation of NLRP3 against Mtb. However, whether epigenetic mechanisms participated in NLRP3 activation is still little known. Here we showed that NLRP3 activation was regulated by DNA methylation modification. Mtb infection promoted NLRP3 activation and inflammatory cytokines expression. NLRP3 promoter was cloned and subsequently identified by Dual-Luciferase Reporter System. The results showed that NLRP3 promoter activity was decreased after methylation by DNA methylase* Sss* I* in vitro*. Meanwhile, DNA methyltransferases inhibitor DAC could upregulate the expression of NLRP3. Furthermore, promoter region of NLRP3 gene was demethylated after Mtb H37Rv strain infection. These data revealed that DNA methylation was involved in NLRP3 inflammasome activation during Mtb infection and provided a new insight into the relationship between host and pathogens.

## 1. Introduction

*Mycobacterium tuberculosis* (Mtb) is one of the leading causes of morbidity and mortality, because about one-third of the world's population is infected with Mtb. According to WHO reports, about several millions of people develop tuberculosis and 1-2 million people die from the disease annually [1].

Mtb infection activates inflammasome in macrophages and dendritic cells [2]. Inflammasome, a multiprotein complexes, is crucial for activation of caspase-1 and generation of mature IL-1*β*, which is important for host immune defense and pathogen elimination [3, 4]. However, the interaction of Mtb infection and inflammasome is very complex [5].

It has been reported that several NOD-like receptors (NLR) are capable of forming inflammasome in response to pathogen associated molecular pattern (PAMP), which triggers activation of NLR and subsequent formation of active inflammasome [6]. NLRP3, a special NLR member, is a cytosolic sensor of danger signals and initiates inflammatory response. The NLRP3 inflammasome is the most characterized inflammasome, which consists of the NLRP3 scaffold, the apoptotic speck protein containing a caspase recruitment domain (ASC) adaptor, and caspase-1 [7]. Currently, researchers find that NLRP3 inflammasome is associated with many kinds of diseases including infectious disease, immune disorders, and Alzheimer's disease [8, 9].

Up to now, much attention has been paid to the mechanisms of NLRP3 regulation against Mtb. The Esx-1 secretion system, a major virulence determinant of mycobacteria, promotes NLRP3 inflammasome activation* in vivo* and* in vitro* [10]. ESAT-6, a 6 kD early secretory antigenic target, is a potent positive regulator of the NLRP3 inflammasome and promotes necrosis in human macrophages [11, 12]. Contrastingly, nitric oxide suppresses NLRP3 inflammasome dependent processing of IL-1*β* and controls the immunopathology of tuberculosis infection [13]. Jin et al. have identified leucine-rich repeat Fli-I interacting protein 2 (LRRFIP2) as an inhibitor of NLRP3 inflammasome activation [14].

DNA methylation, an important epigenetic modification, plays critical roles in key biological processes. Many studies showed that DNA methylation occurred in host genes regulation during viral or bacterial infection, such as Epstein-Barr virus, influenza virus, and* E. coli* [15–17]. In previous study, we found that the expression of IL-6 was regulated by DNA methylation in response to influenza virus infection or dsRNA treatment [18]. In an epidemiologic study, Andraos et al. reported that vitamin D receptor gene methylation is associated with tuberculosis [19]. In addition, Chen et al. found that aberrant methylation of CpG sites in TLR2 promoter region is relevant to active pulmonary tuberculosis [20]. Similarly, Zhang et al. also reported that genotype-specific methylation of polymorphisms in the IL18R1 promoter is attributed to the susceptibility to tuberculosis among Chinese [21]. All these results imply that DNA methylation may be a general modification in host immune response against Mtb infection. However, whether the activation of NLRP3 is regulated by DNA methylation upon Mtb infection remains unclear.

Based on these observations, we hypothesized that the activation of NLRP3 was regulated by DNA methylation. To prove this hypothesis, we investigated the potential epigenetic mechanisms involved in NLRP3 activation during Mtb infection. We demonstrated that the activation of NLRP3 was modulated through NLRP3 promoter demethylation in response to Mtb infection, revealing that DNA methylation was associated with the host gene expression during Mtb infection.

## 2. Materials and Methods

### 2.1. Bacterial Strains

The wild-type* Mycobacterium tuberculosis* H37Rv was used in this study. H37Rv strains were grown in 7H9 broth supplemented with 0.2% glycerol, 0.05% Tween-80, and 10% OADC (Difco).

### 2.2. Cells

The human acute monocyte leukemia cell line THP-1 was maintained in RPMI 1640 (GIBCO) supplemented with 10% fetal bovine serum (Hyclone). The human embryonic kidney cells HEK293T were grown in DMEM medium (GIBCO) containing 10% fetal bovine serum. All cells were incubated at 37°C with 5% CO_2_.

### 2.3. Luciferase Report Vectors Construction

The 1421 bp and 2181 bp fragments of the human NLRP3 gene promoter were inserted into the firefly luciferase vector pGL3-basic (Promega) and termed pNLRP3-P1 and pNLRP3-P2, respectively. The* Renilla* luciferase report vector pRL, an internal control, was purchased from Promega. All constructions were identified by double enzymes digestion and DNA sequencing (Invitrogen, China).

### 2.4. *Mycobacterium tuberculosis* Infection

The THP-1 cells were plated at a density of 2.0 × 10^7^ cells in a 10 cm dish and treated with 20 ng/mL PMA (Phorbol 12-myristate 13-acetate, Sigma) for 48 h. Then, the medium that contained PMA was replaced with fresh RPMI 1640 and cells were infected with Mtb H37Rv at a MOI (multiplicity of infection) of 10 : 1 (Mtb : cells). After 4 h incubation at 37°C, cells were washed three times with RPMI 1640 to remove extracellular bacteria and cultured with RPMI 1640 that contained 10% fetal bovine serum for indicated times.

### 2.5. ELISA

1 mL of the cell culture medium was collected and centrifuged at 12,000 ×g, 4°C for 10 min. All the samples were filtered with 0.22 *μ*m PES membrane (Millipore). The cytokines products were measured using an enzyme-linked immunoassay kit according to protocol (eBioscience).

### 2.6. RNA Extraction and cDNA Synthesis

Total RNA was extracted from Mtb infected or uninfected THP-1 cells using the Trizol Reagent (Invitrogen). Briefly, 2 *μ*g RNA was treated with RNase-free DNase I (Takara) to eliminate genomic DNA contamination. cDNA was synthesized by the SuperScript III First Strand kit following a standard method (Invitrogen) and stored at −80°C until used.

### 2.7. Quantitative Real-Time PCR

Quantitative real-time PCR was carried out using SYBR Premix Ex Taq (Takara) and appropriate primers (Table 1). Fluorescence was monitored by the ABI QuantStudio Flex 6 machine as per the following program: 95°C 30 sec and 40 cycles of 95°C 10 sec, 60°C 8 sec, and 72°C 25 sec, followed by melting curve analysis. Data were normalized to GAPDH according to the 2^(−ΔΔCt)^ method for comparative analysis. Mock or uninfected control was arbitrarily designed as 1. Result was expressed as the mean ± SD of triplicate determinations of a representative experiment. Student's *t*-test was used to determine statistical significance. *p* values <0.05 were considered statistically significant.

### 2.8. Luciferase Reporter Assays

The HEK293T cells were plated at a density of 2.0 × 10^5^ cells per well in 48-well plates and grown to 70% confluence at the time of transfection. The firefly luciferase reporter plasmid pNLRP3-P1 or pNLRP3-P2 was transiently transfected into cells together with the* Renilla* luciferase reporter plasmid pRL using Lipofectamine 2000 Reagent (Invitrogen). Firefly and* Renilla* luciferase activities were measured using Dual-Luciferase Reporter Assay System (Promega) at 48 h after transfection. Firefly luciferase activity was normalized to* Renilla* luciferase activity, and the promoter activity was calculated as relative luciferase activity. The NLRP3 promoter relative activity was expressed as the mean ± SD of triplicate or quadruplicate wells of a representative experiment performed three times. Student's *t*-test was used to determine statistical significance. *p* values <0.05 were considered statistically significant.

### 2.9. DNA Methylation* In Vitro*

1 *μ*g of pNLRP3-P1 or pNLRP3-P2 was treated with S-adenosylmethionine and* Sss* I, a DNA methylase (New England Biolabs) at 37°C for 4 h; meanwhile, 1 *μ*g of plasmid was incubated similarly but without* Sss* I methylase (unmethylated control). Then, all the plasmids were purified with PCR product clean-up kit (Axygen). The unmethylated or methylated promoter activities were measured as per the above methods.

### 2.10. MTT

Cytotoxicity assay was detected using Cell Counting Kit-8 (Dojindo, Japan). 10 *μ*L of CCK-8 solutions was added to 100 *μ*L cell culture medium. After incubation at 37°C for 4 h, the absorbance at 450 nm was measured with Synergy2 machine (BioTek).

### 2.11. Sodium Bisulfate Sequencing

Genomic DNA of THP-1 cells infected with Mtb or not was extracted using AxyPrep Genomic kit (Axygen). Sodium bisulfate sequencing was preformed using EZ DNA Methylation Gold Kit (Enzo). Briefly, 2 *μ*g of genomic DNA was treated according to the manufacturer's instructions. The modified DNA was amplified with primers (Table 1) specific for the NLRP3 promoter region. The PCR products were purified and then inserted into pMD18T vector (Takara). The DNA methylation status was analyzed with sequencing data, which was checked using a standard BiQ Analyzer software.

### 2.12. Illumina Infinium 450K Array Analysis

Illumina Human Methylation 450 Bead Chip (Illumina) was used to detect whole genome methylation status. Genomic DNA of THP-1 cells infected with Mtb or not was extracted using Genomic kit (Qiagen) and bisulfite-converted using the EZ DNA Methylation Kit (Zymo). Converted DNA was hybridized to Infinium Human Methylation 450 Bead Chip. The following analysis was provided by Genergy, Co. (Shanghai, China).

## 3. Results

### 3.1. Mtb Infection Promotes NLRP3 Activation and Inflammatory Cytokines Secretion

**Figure 1:**
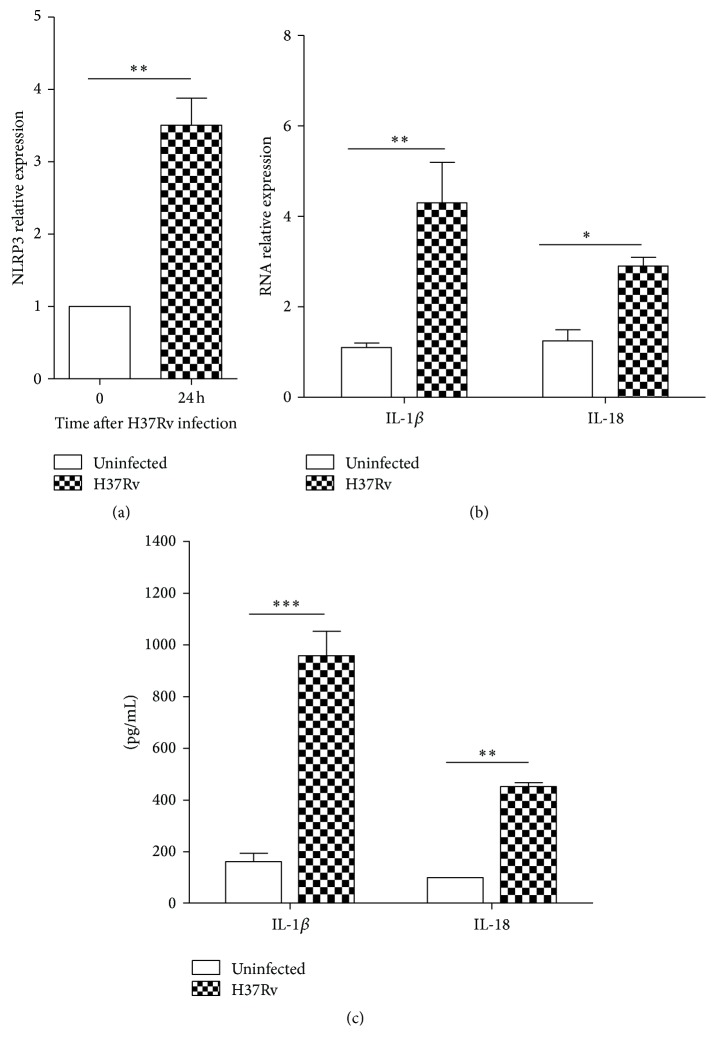
Mtb promoted NLRP3 activation and inflammatory cytokines expression. The THP-1 cells were plated in 10 cm dish and treated with 20 ng/mL PMA for 48 h; then the cultural media that contained PMA were replaced with fresh RPMI 1640 before infection. MOI was 10 : 1 in all experiments. NLRP3 (a) and inflammatory cytokines (b) mRNA relative expression levels were detected by quantitative real-time PCR 24 h after H37Rv infection. Data were normalized to housekeeping gene GAPDH according to the 2^(−ΔΔCt)^ method. (c) Production of inflammatory cytokines was measured by ELISA 48 h after H37Rv infection (^*∗*^ *p* < 0.05, ^*∗∗*^ *p* < 0.01, and ^*∗∗∗*^ *p* < 0.001).

To verify NLRP3 expression during Mtb H37Rv infection, the transcription level of NLRP3 was analyzed by real-time quantitative RT-PCR. As expected, the mRNA transcript level of NLRP3 was upregulated about 3 times 24 h after Mtb infection (Figure 1(a)).

Then, mRNA levels of NLRP3 inflammasome related cytokines were measured. mRNA relative expressions of IL-1*β* and IL-18 were upregulated 24 h after Mtb infection (Figure 1(b)). Meanwhile, we test the inflammatory cytokines production by ELISA. Results showed that IL-1*β* and IL-18 were sharply increased after Mtb infection (Figure 1(b)). These results verified that Mtb infection induced NLRP3 activation and inflammatory cytokines release from THP-1 cells, so this infection model was used in a following study for DNA methylation modification mechanisms.

### 3.2. Construction and Identification of NLRP3 Promoter

**Figure 2:**
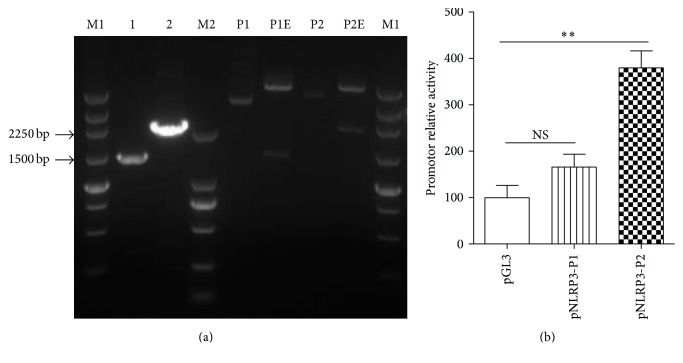
Construction and identification of NLRP3 promoter. (a) Two potential promoter regions of NLRP3 were amplified from genome and identified by double restriction enzyme digestion. 1 and 2 were PCR products from human genomic DNA. P1 and P2 were pNLPR3-P1 and pNLPR3-P2, respectively. P1E and P2E exhibited the enzyme digested products of pNLPR3-P1 and pNLPR3-P2 digested by* Xho* I and* Mlu* I. M1 and M2 were 250 bp and DL2000 DNA marker, respectively. (b) The activities of two potential NLRP3 promoters were identified by Dual-Luciferase Reporter Assay. HEK293T cells were plated in 24-well cell culture plate and cotransfected with firefly luciferase report plasmids pNLRP3-P1/P2 and* Renilla* luciferase report plasmid pRL. The relative promoter activity was measured 48 h after transfection as in Materials and Methods. Firefly luciferase activity was normalized to* Renilla* luciferase activity, and the promoter activity was expressed as the mean ± SD of at least triplicate wells (^*∗∗*^ *p* < 0.01; NS: no significant difference).

To gain more insight into the epigenetic mechanisms involved in NLRP3 activation, two potential promoter regions of NLRP3 were predicted and amplified from human genome DNA by PCR. Then, these two amplified fragments were inserted into firefly luciferase reporter plasmid pGL3-basic.  Figure 2(a) showed the PCR product of two fragments and the results of double restriction enzymes digestion. Two constructed plasmids were also identified by DNA sequencing and termed as pNLRP3-P1 and pNLRP3-P2, respectively (data not shown).

We preformed the luciferase reporter assay to evaluate the activities of the two promoters. HEK293T cells were cotransfected with pNLRP3-P1 or pNLRP3-P2 and pRL. Cells transfected with pGL3-basic and pRL were used as control. Significant luciferase activity was observed when pNLRP3-P2 was transiently transfected into the HEK293T cells, so we used it as NLRP3 promoter in the following study (Figure 2(b)).

### 3.3. NLRP3 Promoter Activity Regulated by DNA Methylation

**Figure 3:**
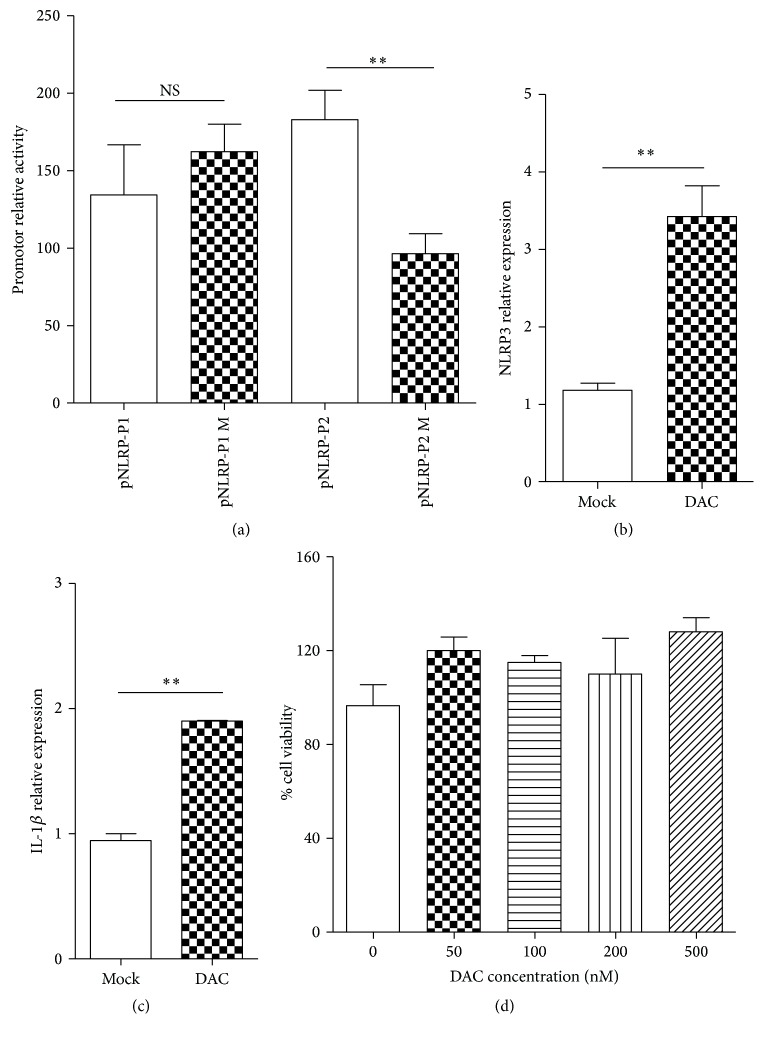
DNA methylation modification regulated NLRP3 activation* in vitro*. (a) The methylated (*Sss* I treated) or unmethylated pNLRP3-P1/P2 was transfected into HEK293T cells together with pRL, respectively, and each relative promoter activity was measured 48 h after transfection. The mRNA relative expression levels of NLRP3 (b) and IL-1*β* (c) in PMA stimulated THP-1 cells were detected 48 h after treatment with 100 nM DAC. (d) Supernatants of PMA stimulated THP-1 cells were collected 48 h after treatment by different concentrations of DAC, and the cell viability was analyzed using CCK-8 kit (^*∗∗*^ *p* < 0.01; NS: no significant difference).

The NLRP3 expression level is considered possessing vital position in inflammasome activation, so the mechanisms of the expression modulate have been extensively studied. Here, we aimed to elucidate whether DNA methylation directly influenced NLRP3 activation. The NLRP3 promoter activity was investigated after methylation* in vitro*. The plasmids of pNLRP3-P1 and pNLRP3-P2 were methylated by* Sss* I* in vitro* before being transfected into HEK293T cells. The result demonstrated that, compared with the unmethylated promoter, the activity of methylated pNLRP3-P2 decreased to 50%, but pNLRP3-P1 promoter activity showed no significant difference (Figure 3(a)). These results implied that DNA methylation alteration might be involved in the regulation of NLRP3 promoter and further suggested that pNLRP3-P2 contained the potential activated promoter region of NLRP3.

To further investigate the role of DNA methylation modification in NLRP3 inflammasome activation, the mRNA levels of NLRP3 and inflammatory cytokine IL-1*β* were detected after DNMT (DNA methyltransferase) inhibitor treatment. THP-1 cells stimulated with PMA were treated with 100 nM DAC (5-aza-2′-deoxycytidine) for 48 h; then total RNA was extracted and cultural supernatants were collected. As shown in Figures 3(b) and 3(c), mRNA transcript levels of NLRP3 and IL-1*β* were upregulated about 2.7 times and 2 times, respectively, after DAC treatment. Meanwhile, MTT assay was carried out to test the influence of cell ability. The result demonstrated that treatment with different amounts of DAC had no effect on cell proliferation and viability (Figure 3(d)). These results firstly implied that DNA methylation modification was involved in NLRP3 activation in artificial mimic instead of in physiological manner.

### 3.4. Methylation Status of the NLRP3 Promoter Region during Mtb Infection

**Figure 4:**
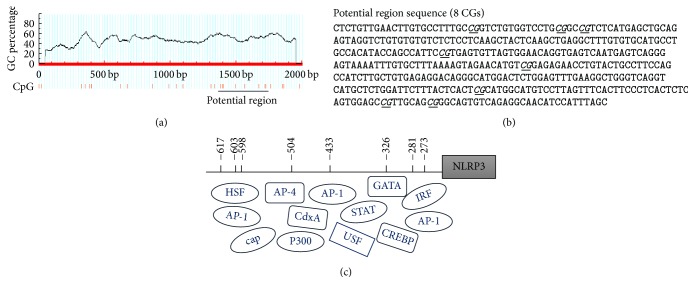
Analysis of NLRP3 promoter region. (a) Computational prediction of CpG-rich sites in NLRP3 promoter. Criteria used for prediction were island size >200 bp, GC percentage >50%, and observed/expected CpG ratio >0.6. (b) Sequence of potential promoter region. CpG sites were underlined and italic. (c) Schematic overview of CpG sites located in NLRP3 promoter region. The boxes of transcript factors matched their binding sites in promoter region. The binding sites were predicted using TFSEARCH software.

The pNLRP3-P2 spanning from position −2030 bp to +151 bp was analyzed through MethPrimer, which is a CpG island prediction program (http://www.urogene.org/methprimer/). The result showed that this fragment had several CpG-rich site regions (Figure 4(a)). Interestingly, we found that NLRP3 promoter region from position −650 bp to −200 bp contained many binding sites for active transcription factors, such as CRE-BP, AP-1, and HSF (Figures 4(b) and 4(c)). Therefore, we analyzed the methylation status of this region next.

**Figure 5:**
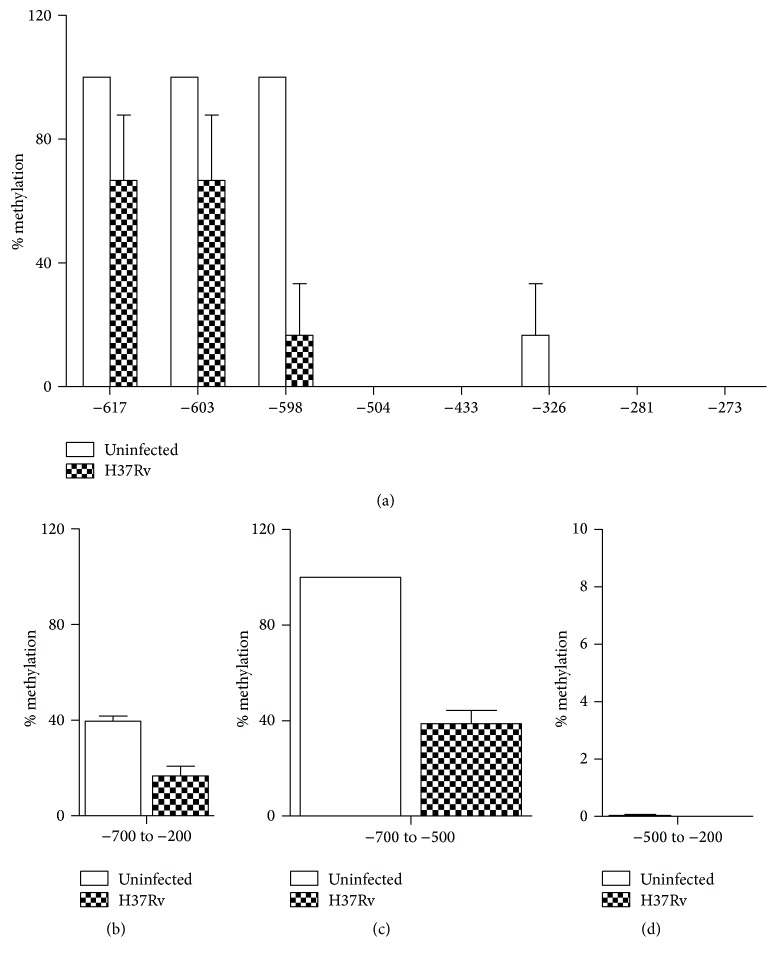
Methylation levels of NLRP3 promoter region during Mtb infection. Genomic DNA of THP-1 cells was extracted 24 h after Mtb infection. (a) Methylation status of CpG sites was measured by direct sodium bisulfate DNA sequencing as in Materials and Methods. Methylation status of −700 to −200 (b), −700 to −500 (c), and −500 to −200 (d) regions was analyzed. At least 10 clones were sequenced for each group.

The methylation percentage of genomic DNA extracted from THP-1 cells infected by Mtb H37Rv or not was quantified by sodium bisulfate sequencing. As shown in Figure 5(a), we found that the region from −700 bp to −200 bp was hypomethylation in two groups (16.7% versus 39.3%), especially in the region from −500 bp to −200 bp which was all unmethylated. However, in the region from position −700 to −500 bp, the methylation level was notably lower in Mtb infected group (44%) than that in uninfected group (100%) (Figures 5(b)–5(d)). This result indicated that demethylation of the NLRP3 promoter region occurred upon Mtb infection, which may influence the expression of NLRP3 gene and activation of inflammasome.

**Figure 6:**
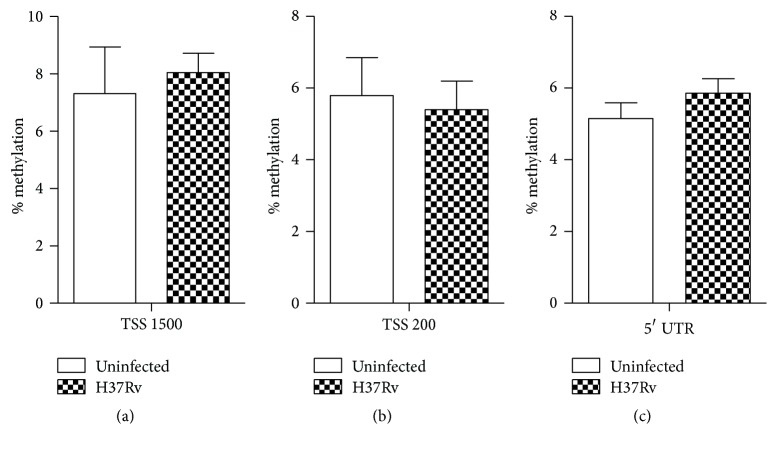
Methylation status of GAPDH promoter region during Mtb infection. Genomic DNA of THP-1 cells was extracted 24 h after Mtb infection or not. The methylation levels of TSS 1500 (a), TSS 200 (b), and 5′ UTR (c) were detected by Illumina Infinium 450K bead arrays. TSS stands for transcription start site of NLRP3.

To confirm the specificity of NLRP3 promoter methylation alternation in Mtb infection, methylation status of housekeeping gene GAPDH promoter was analyzed by Illumina Infinium 450K Bead Chip. We found that the methylation levels of 5′ UTR, 1500 bp, and 200 bp upstream of transcription start site were all not significantly different after Mtb infection (Figure 6). These furtherly proved that DNA methylation could modify NLRP3 promoter and then influence NLRP3 activation.

## 4. Discussion

This study provided evidence for the notion that NLRP3 activation promoted by* Mycobacterium tuberculosis* was regulated by DNA methylation modification. It indicated that the expression of host gene was affected by epigenetic modification during Mtb infection.

Previous reporters have showed that the global host immune response is activated against Mtb infection [5]. In this process, NLRP3 is an important NLR which recognize PAMPs of Mtb and then activate ACS and caspase-1 pathway, resulting in proinflammatory cytokines secretion, such as IL-1*β* and IL-18. Meanwhile, several negative regulators of NLRP3 have been reported. The LRRFIP2 can bind to NLRP3 and subsequently inhibit caspase-1 pathway [14]. NO (nitric oxide) which is increased during Mtb infection has the capacity to negatively regulate the NLRP3 inflammasome so that it can suppress tissue damage produced by continues activation of innate immunity and control the immunopathology of tuberculosis [13, 22, 23]. Furthermore, Yan et al. proposed that omega-3 fatty acid, another negative regulator of the NLRP3 inflammasome, prevents excessive inflammation and metabolic disorder [24]. They also reported that dopamine suppresses NLRP3 inflammasome activation to control systemic inflammation [25]. All these findings demonstrate that NLRP3 inflammasome regulatory pathways during Mtb infections are complex. Given the critical roles of NLRP3, we focus on regulation of NLRP3 activation during Mtb infection in this study. As shown in Figure 1, we verified that NLRP3 was initiated upon Mtb infection, and then mature inflammatory cytokines were produced.

This is now general consensus that NLRP3 activation is targeted by certain molecules of Mtb, such as ESAT6 [12, 26]. However, few studies focus on the epigenetic mechanisms of mediated NLRP3 regulation. Methylation of CpG sites, especially at promoter regions, is a key and conserved epigenetic modification in regulating gene expression in many species [27]. Therefore, we hypothesized that DNA methylation may be involved in NLRP3 activation during Mtb infection. To elucidate this hypothesis, we investigated the effect of DNA methylation on NLRP3 activation. The results showed that the NLRP3 promoter activity was inhibited after being methylated* in vitro* with DNA methylase* Sss* I, and the transcriptional level was increased by treatment with DNMT inhibitor DAC (Figure 3). These indicated that DNA methylation regulated expression of NLRP3 in artificial mimic manner and also implied that it might also play roles in physiological level.

DNA methylation has a profound impact on genome stability and gene expression [27]. Hypomethylation of the gene promoter region is a feature of activation, whereas hypermethylation is associated with gene silencing. Bisulfite sequencing assay showed that, despite the fact that the NLRP3 promoter region from −700 bp to −200 bp was hypomethylated upon Mtb infection or not, the region from −700 to −500 bp was demethylated after Mtb infection (Figure 5), supporting the hypothesis that methylation of the CpG sites of promoter region plays a role in NLRP3 activation during Mtb infection. In addition, methylation level of the housekeeping gene GAPDH promoter was not significantly different after Mtb infection (Figure 6), further proving that NLRP3 promoter was specifically regulated by DNA methylation modification.

Generally, DNA methylation regulates gene expression through direct or indirect way [28]. The direct way interferes with the recruitment and binding of transcription factors to the gene promoter regions [18], while the indirect way occupied the methylated promoters regions and competed for the transcription factor binding sites through recruiting sequence-independent methylated DNA-binding proteins, such as MeCP2 and MBD [29–31]. Our study indicated that the modulation of NLRP3 gene expression may be via the direct way, because the promoter region from −700 to −500 bp, where methylation status has changed, owns many transcription factor binding sites in DNA sequence. Moreover, change of methylation status may influence chromatin structure of special regions and recruit regulators. The binding of transcription factor to promoter region will be intensively investigated using EMAS or ChIP methods in our further study.

In conclusion, our study has researched the mechanism of NLRP3 promoter methylation associated with NLRP3 activation during* Mycobacterium tuberculosis* infection. In host cells, NLRP3 gene was silenced because of the hypermethylation of promoter. Mtb infection resulted in the demethylation of the NLRP3 promoter region. The decrease of methylation level at promoter region participated in activation of NLRP3. This study not only revealed the epigenetic mechanisms of NLRP3 inflammasome activation but also provided new insights into understanding the regulation mechanism underlying Mtb infection and interaction between pathogens and host.

## Figures and Tables

**Table 1:** Primers used in this study.

Primer	Sequence (5′-3′)	Purpose
GAPDH-F	AAGGTGAAGGTCGGAGTCAAC	GADPH qPCR
GAPDH-R	GGGGTCATTGATGGCAACAATA	

NLRP3-F	GGAGAGACCTTTATGAGAAAGCAA	NLRP3 qPCR
NLRP3-R	GCTGTCTTCCTGGCATATCACA	

IL1*β*-F	CAAGGGCTTCAGGCAGGCCG	IL-1*β* qPCR
IL1*β*-R	TGAGTCCCGGAGCGTGCAGT	

Methyl-F	TTTTGTTGAATTTGTGTTTTTGT	Sodium bisulfate
Methyl-R	ACTAAATAAATATTACCTCTAACACTA	
